# Comparative Study of Human Age Estimation with or without Preclassification of Gender and Facial Expression

**DOI:** 10.1155/2014/905269

**Published:** 2014-09-09

**Authors:** Dat Tien Nguyen, So Ra Cho, Kwang Yong Shin, Jae Won Bang, Kang Ryoung Park

**Affiliations:** Division of Electronics and Electrical Engineering, Dongguk University, Seoul 100-715, Republic of Korea

## Abstract

Age estimation has many useful applications, such as age-based face classification, finding lost children, surveillance monitoring, and face recognition invariant to age progression. Among many factors affecting age estimation accuracy, gender and facial expression can have negative effects. In our research, the effects of gender and facial expression on age estimation using support vector regression (SVR) method are investigated. Our research is novel in the following four ways. First, the accuracies of age estimation using a single-level local binary pattern (LBP) and a multilevel LBP (MLBP) are compared, and MLBP shows better performance as an extractor of texture features globally. Second, we compare the accuracies of age estimation using global features extracted by MLBP, local features extracted by Gabor filtering, and the combination of the two methods. Results show that the third approach is the most accurate. Third, the accuracies of age estimation with and without preclassification of facial expression are compared and analyzed. Fourth, those with and without preclassification of gender are compared and analyzed. The experimental results show the effectiveness of gender preclassification in age estimation.

## 1. Introduction

With the development of smart devices, such as smart phones and smart televisions, natural user interfaces (NUIs) become increasingly attractive. In addition, with the vigorous research on three-dimensional (3D) video processing techniques on 3DTV [1], 3DTV NUIs can be also considered. NUIs offer the advantage of natural interaction with a system using predefined actions and/or physical human characteristics. For example, people have been using hand gestures, speech [2], biosignals [3], and mobile device gestures [4] to interact with machines such as computers and smart devices. In previous research [5], finger-triggered virtual musical instruments have also been proposed based on a finger data-glove system. Along with gesture and speech, the human face, which contains substantial information about a person, has also been widely used for human-machine interaction in many applications including face-based human identification, gender classification, age estimation, facial expression recognition, and race classification. Among these, age estimation using facial images is becoming increasingly important. In previous studies [6–10], age synthesis and estimation have been studied with many real-world applications, such as forensic art, surveillance monitoring, biometrics, cosmetology, and entertainment. In addition, age estimation is used for face recognition invariant to age progression [11, 12]. Because of the changing of facial characteristics, such as facial shape and skin detail, according to age progression, face recognition performance is less effective if it neglects age effects. In this case, age estimation can serve as a complement to the primary biometric feature of face. The age estimation and synthesis have been also used for finding lost children. Using age estimation and synthesis, the updated face appearance of lost children after several years can be predicted. Age estimation can be used to prevent children from accessing adult websites and restricted videos and from buying tobacco from automated vending machines.

Previous age estimation methods based on facial images can be divided into two categories: active appearance model- (AAM-) based and non-AAM-based methods [13]. AAMs are statistical face models and have been used for age estimation; they involve the modeling of face's shape and appearance [6–8]. With a training data set, models of face shape and appearance are generated based on multiple feature points. Then, age is considered as a function of feature vectors learned from AAMs. Although the AAM-based method can produce high age estimation accuracy, its performance is strongly affected by the problem of fitting multiple feature points. Further, it involves substantial processing time to locate the feature points. Thus, the AAM-based method is difficult to use in real-time systems.

Another approach to age estimation is non-AAM-based methods. Choi et al. used the high frequency components of selected skin regions for age estimation [9]. In this study, the high frequency components are measured by using high-pass filters, such as a Sobel filter, image differences between an original and its smoothed images, an ideal high-pass filter (IHPF), a Gaussian high-pass filter (GHPF), and wavelet transforms. The work in [10] used the local binary pattern (LBP) operator to extract age features from skin regions of a face. Although these approaches can be used for age estimation, they still have limitations. The estimation performance of the work in [9] is strongly dependent on the method of choosing skin regions (where the frequency components are extracted), which are determined by the facial feature points. The performance enhancement of the work in [10] is limited by the use of a single-level LBP operator. To overcome this problem, the work in [13] proposed an age estimation method based on a multilevel LBP (MLBP) and support vector regression (SVR). However, they did not consider the local features for age estimation.

Based on human perception, we can see that there are some differences between men and women in terms of producing facial age features. For example, an adult man can have a beard and rough skin surface, whereas a woman does not have a beard and tends to have smoother skin compared to a man. This suggests that gender can have effects on age estimation. In previous studies, gender is recognized by voice, 3D body shape, or face image. In different ways, they show that gender recognition accuracy is affected by age.

Another factor that can produce negative effects on age estimation accuracy is facial expression. Humans of the same age can show different age features of textures, among different facial expressions. There have been many previous studies of facial expression recognition based on both the shape and/or texture appearance of a face image. In different aspect, they showed the effect of age on facial expression recognition performance.

Most of previous researches did not consider the effect of facial expression and gender on the age estimation system. Considering the limitations of previous research, we propose a new age estimation method. In addition, the effects of gender and facial expression on age estimation are investigated. The accuracies of age estimation using LBP and MLBP are compared, and MLBP shows better performance as the extractor of texture features globally. In addition, we compare the accuracies of age estimation using global features extracted by MLBP, local features extracted by Gabor filtering, and the combination of the two methods. Results showed that the third approach is superior. In addition, the accuracies of age estimation with and without preclassification of facial expression and gender are compared. We implemented an enhanced age estimator compared to that of [13], and the novelties of our research (as shown in Abstract) are different from those in [13].

The remainder of this paper is organized as follows. In Section 2, an overview of age estimation system is provided. In Section 3, experiments are conducted using the proposed method with PAL aging database to determine the effectiveness of the proposed method and verify the effects of gender and facial expression on age estimation. Based on the experimental results, we discuss the effects of gender and facial expression on age estimation in Section 4. Finally, conclusions are presented in Section 5.

## 2. Age Estimation System: Overview

### 2.1. Overview of Proposed Age Estimation System

**Figure 1:**
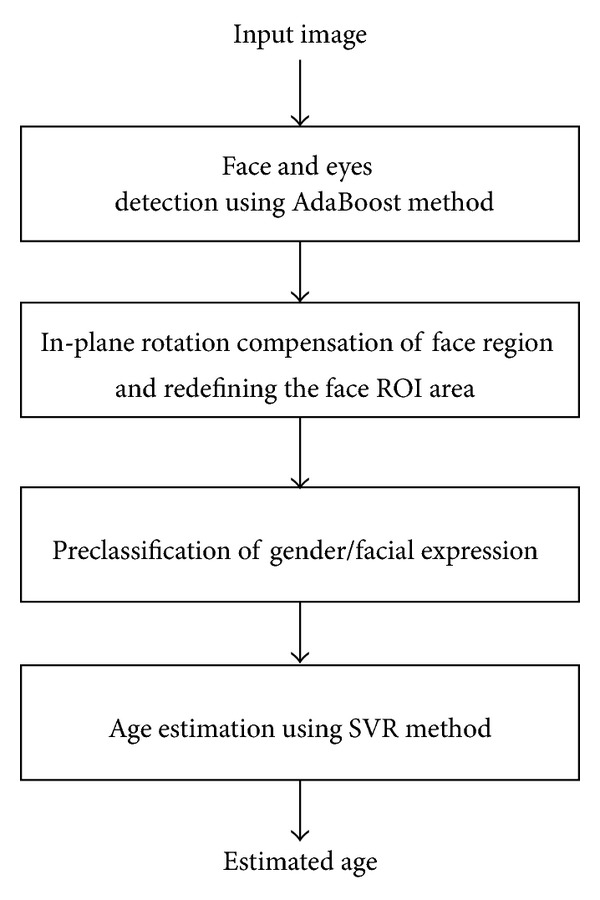
Overview of an age estimation system that considers the effects of gender and facial expression.

An overview of our age estimation system, which considers the effects of gender and/or facial expression, is depicted in Figure 1. For the preprocessing step, the face and eye positions are first detected from the input image using adaptive boosting (AdaBoost) method [13, 14]. Then, our system conducts in-plane rotation to align the face based on the detected positions of the two eyes [13].

As shown in Figure 1, to consider the effects of gender or facial expression on age estimation, preclassification of gender/facial expression is conducted before age estimation. The gender or facial expression preclassification procedure allows us to deal with each gender (male and female) and facial expression (neutral, happy, surprised, and the like) separately for age estimation. In our research, the automatic gender and facial expression recognition algorithm is not implemented but is left for future work. For the initial research, we divide the experimental data manually according to gender and facial expression to measure the effects of gender or facial expression on age estimation. In our experiments, we compared the accuracies of age estimation without preclassification of gender and facial expression, with preclassification of gender, and with preclassification of facial expression. Finally, our system estimates the age of an input face using SVR [13].

### 2.2. Face Detection and In-Plane Rotation Compensation

Typically, an input facial image contains both the facial and background regions. Therefore, the first step of the age estimation system is to localize the facial region in the input image. There have been many previous studies of face detection [15–17], and we use the AdaBoost method [13, 14]. With AdaBoost, the facial region and the positions of the eyes could be detected efficiently by constructing a strong facial classifier from several weak facial classifiers. In the actual system, there typically exists an in-plane face rotation in the captured image, which degrades the performance of the age estimation system. Previous work [18] proved that age estimation systems could be affected by misalignment of the face region. Thus, we use the detected positions of the eyes to compensate for the in-plane rotation of the face [13]. In detail, within the detected face region, the positions of right and left eyes are located as (*R*_*x*_, *R*_*y*_) and (*L*_*x*_, *L*_*y*_), respectively. Then, the angle of in-plane rotation is calculated by (1), and the input face region is rotated by the angle *θ*:

(1) $$\begin{array}{c} \theta =\text{ta}{\text{n}}^{-1}\left(\frac{R_{y}-L_{y}}{R_{x}-L_{x}}\right). \end{array}$$

**Figure 2:**
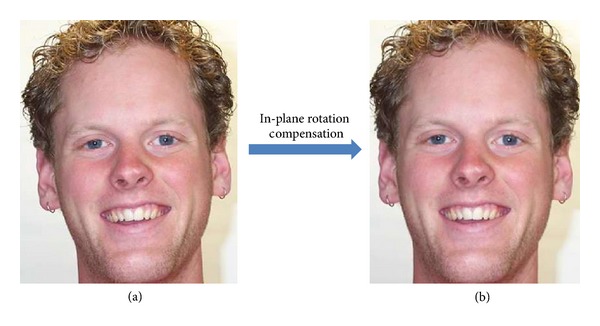
Example of in-plane rotation compensation: (a) an example rotated image and (b) in-plane rotation compensation result of the image in (a).

Figure 2 shows an example of our in-plane rotation compensation method.

**Figure 3:**
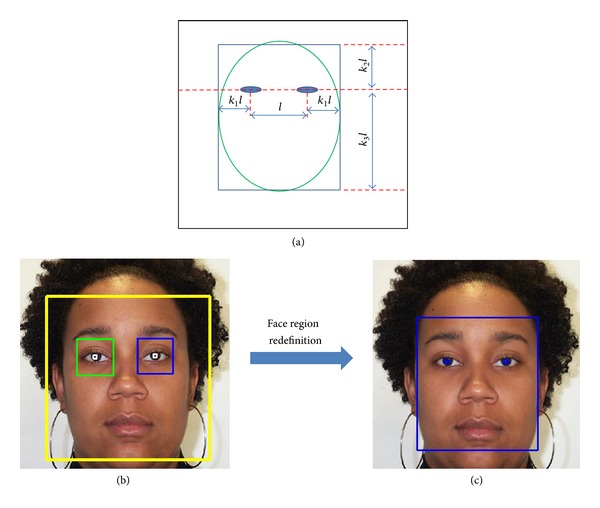
The example of face region redefinition: (a) methodology for face region definition, (b) face region detected using AdaBoost and in-plane rotation compensation, and (c) result of face region of interest (ROI) redefinition.

Although AdaBoost has been widely used to detect a face from a face image, it cannot localize the face region correctly, as shown in Figure 3(b). To estimate the face region more accurately, our system performs a redefinition step based on the positions of the eyes, as shown in Figures 3(a) and 3(c).

Suppose that the distance between the eyes is measured as a value of *l* (pixels) based on the result of detected eye positions and the compensation procedure. Then, our method uses a redefinition method to estimate the face ROI based on the *l* value as shown in Figure 3(a), where *k*_1_, *k*_2_, and *k*_3_ are the ratio values considering face geometry and defined by experiment [13]. In detail, the ratio values (*k*_1_, *k*_2_, and *k*_3_) were experimentally determined so as not to include background and hair regions for age estimation. The determined values of *k*_1_, *k*_2_, and *k*_3_ are 0.5, 0.75, and 1.5, respectively.

### 2.3. The Proposed Method for Human Age Estimation

#### 2.3.1. Age Estimation Based on MLBP, Gabor Filter, and SVR

**Figure 4:**
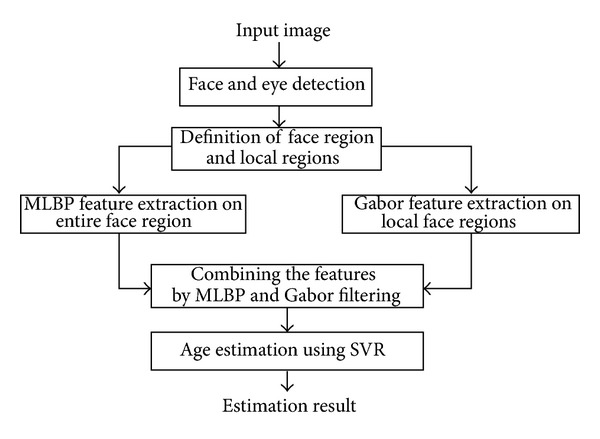
Proposed age estimation method based on MLBP, Gabor, and SVR.

In Figure 4, we show the proposed age estimation method based on the combination of MLBP, Gabor filter, and SVR. There have been many previous studies of local texture analysis [19]. In previous researches, the LBP has been successfully used for facial expression recognition, age estimation, and pattern recognition. However, those studies used only the single level for LBP instead of multilevel for LBP [7, 10]. Therefore, in our proposed method, the MLBP is used to create a stronger descriptor for age estimation.

Along with global features extracted using MLBP, the proposed method also extracts local features for age estimation. The wrinkle feature is a very important feature appearing locally on the human face [7, 9]. Therefore, in our method, the wrinkle feature is used as a local feature for enhancing the age estimation result. For this purpose, we use Gabor filtering [7]. Finally, the feature vector formed by combining the global and local features is used for estimating age using SVR.

#### 2.3.2. Global Feature Extraction Using MLBP

LBP is a powerful method for describing image texture by thresholding the surrounding pixels with a center pixel [7, 10, 13]. The LBP method has been widely used in many researches such as age estimation [7, 10, 13], gender recognition, finger-vein recognition, facial expression recognition, and face recognition. The main advantage of LBP method is that it offers the texture descriptor robustness to the variations of illumination and rotation. Besides, the fast processing can be done by LBP method. The LBP operator is defined as [13]

**Figure 5:**
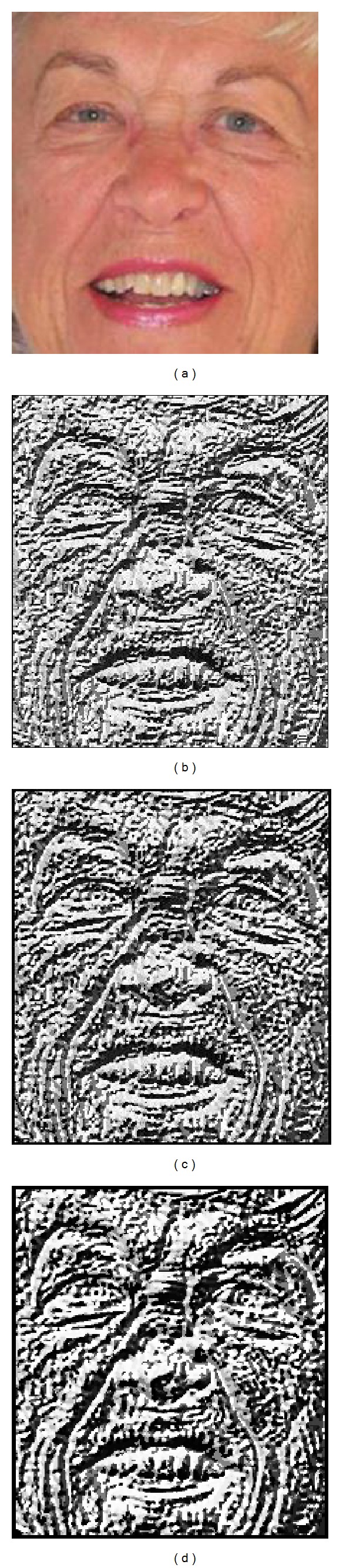
Example of texture extraction by LBP method at different values of *R* and *P*: (a) an example facial image, texture extraction by LBP operator with (b) *R*and *P* of 1 and 8, respectively, (c) *R*and *P* of 2 and 8, respectively, and (d) *R*and *P* of 3 and 12, respectively.

(2) LBPR,P=∑i=0P−1s(gi−gc)2i,where  s(x)={1,if  x≥00,if  x<0,

where *P* is the number of neighboring pixels, *R* is the radius of the LBP circle (the distance from the center pixel to the neighboring pixels), *g*_*i*_ and *g*_*c*_ are the gray level of neighboring pixels and center pixel, respectively, and *s*(*x*) is the threshold function. Varying the values of *R* and *P*, we extract image texture features at different scales and resolutions [13]. Originally, the LBP operator makes the texture descriptor by using a 3 × 3 mask. In this case, the values of *R* and *P* are 1 and 8, respectively. According to the human's age, the features for age estimation appear in different size (bigger/smaller spot, wrinkle, etc.). In addition, the resolution and size of facial image also causes the size difference of the features. Consequently, varying the values of *R* and *P* makes our method obtain the sufficient information of features at different scales and resolution.  Figure 5 shows an example of texture feature of a facial image at different values of *R* and *P*. As shown in Figure 5, while the LBP operator with smaller *R* and *P* values extracts the fine textures of narrow thickness (Figure 5(b)), that with the bigger values helps our method extract the coarse textures of wide thickness.

**Figure 6:**
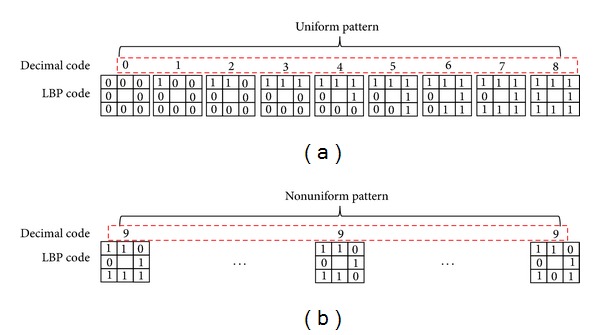
Example of uniform and nonuniform patterns and their assigned decimal codes in the case in which *R* and *P* are one and eight, respectively: (a) uniform patterns and (b) nonuniform patterns.

**Figure 7:**
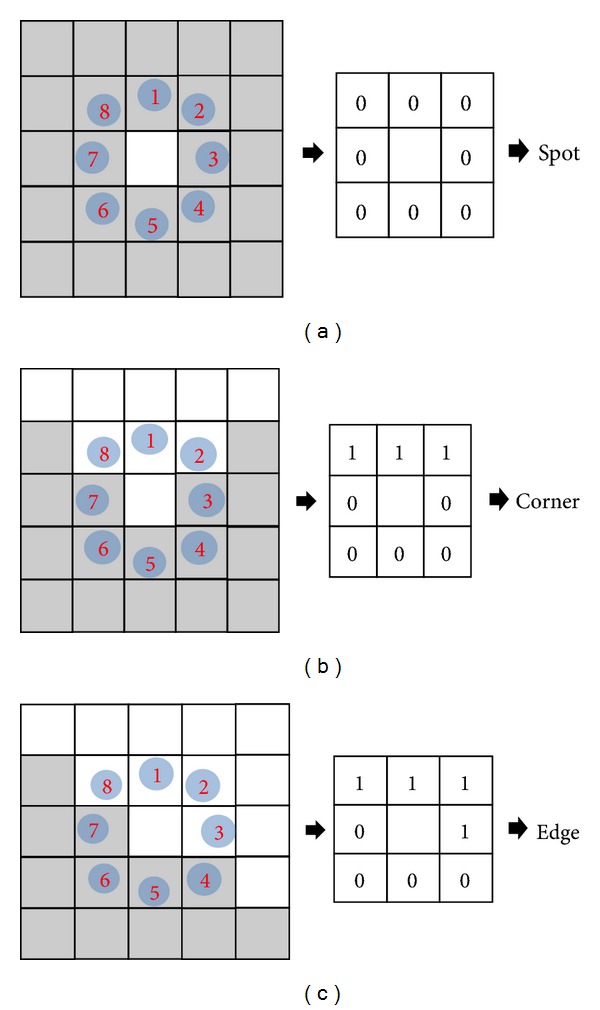
Examples of uniform patterns in describing the texture features: (a) spot feature, (b) corner feature, and (c) edge feature.

The extracted LBP binary codes from (2) are classified into uniform or nonuniform patterns, as shown in Figure 6 [13]. As the human becomes older, several facial texture features such as wrinkle and spot appear [7, 10, 13]. Based on this characteristic, our research uses the LBP operator to describe the image texture and extract the feature for age estimation. A uniform pattern contains at most two bitwise transitions from 0 to 1 (or 1 to 0), as shown in Figure 6(a). Otherwise, a pattern is classified as nonuniform. Uniform patterns are useful for describing image textures, such as spots, corners, and lines, whereas nonuniform patterns contain insufficient information for describing image texture.  Figure 7 shows the examples of representing the textures of spot, corner, and edge into the uniform patterns of 0, 3, and 4 of Figure 6(a), respectively.

Then, our system forms the image descriptor by accumulating the LBP code histogram (uniform or nonuniform code) over the texture. Figure 6 shows an example of uniform and nonuniform codes as well as the assigned codes in the case that *R* and *P* are one and eight, respectively. Uniform codes are assigned decimals from zero to eight, whereas all of the nonuniform codes are assigned the decimal nine. By accumulating the histogram of assigned decimal codes of uniform and nonuniform codes in an image, we form a histogram of texture appearance and use it as the image descriptor in age estimation [7, 10, 13].

**Figure 8:**
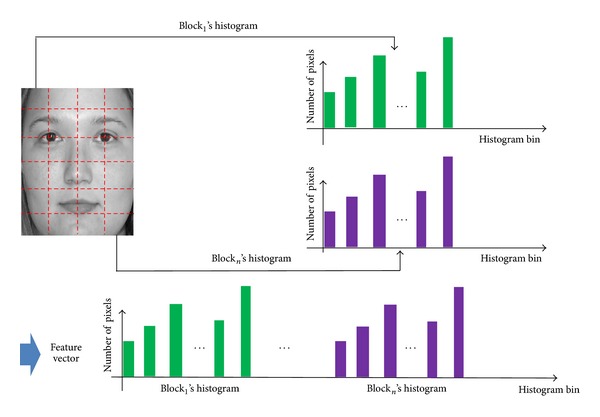
Method for constructing the LBP feature vector from multiple subblocks.

The LBP code histogram represents the distribution of image textures in an image. The LBP features of binary code can represent the more local texture compared to the histogram features. However, the LBP binary code can be much affected by image misalignment, and we use the histogram features in our research. To extract the various features of image texture, the proposed system divides the input image into local subblocks and forms the descriptor (feature vector) for each subblock. Consequently, the final descriptor of the image is formed by concatenating the descriptor of each subblock.  Figure 8 depicts the method for constructing the LBP descriptor of an image. The feature vectors of each subblock are concatenated together as shown in Figure 8. In our research, the order of concatenating the feature vectors of subblocks is from left to right and up to down. That is, the histogram of the upper-left subblock is included first and that of lower-right one is included last in the final LBP feature vector.

**Figure 9:**
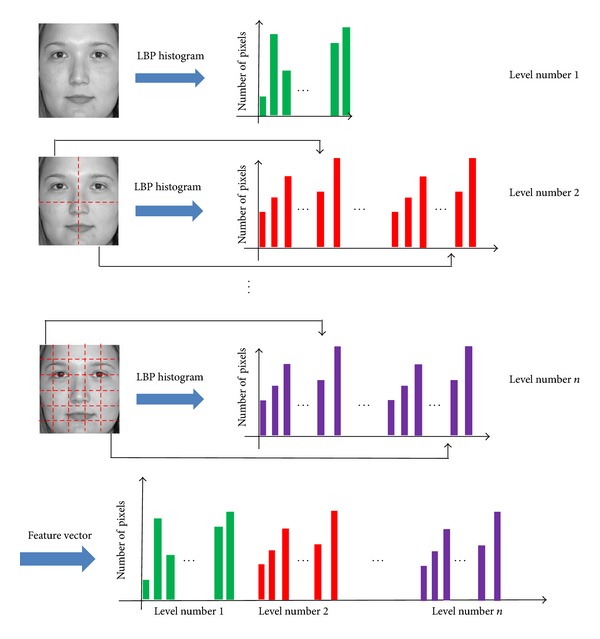
Method for constructing MLBP feature vector from multiple single-level LBP feature vectors.

Based on the single-level LBP method of Figure 8, our method constructs the MLBP features by concatenating the several feature vectors of the single-level LBP, as shown in Figure 9. The accuracy of age estimation by LBP is affected by the size of subblock. With the larger subblock, the global features are extracted by LBP whereas with the smaller one, the local features can be obtained. In order to extract the various features globally and locally, we use the MLBP of Figure 9 instead of single-level LBP of Figure 8. The optimal level (with which the smallest error of age estimation is obtained) is determined experimentally to be three for MLBP [13].

#### 2.3.3. Local Feature Extraction by Gabor Filtering

**Figure 10:**
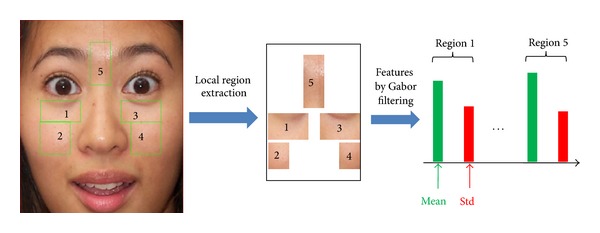
The 5 selected areas for extracting wrinkle features.

In previous work by Choi et al. [7], the wrinkle feature is extracted locally using Gabor filtering at nine specific face regions. The definition of wrinkle areas is based on the 68 facial feature points defined manually or automatically as an AAM fitting result. Although the definition of wrinkle areas based on 68 facial feature points is very accurate, it has the problem that it takes a long processing time to determine the 68 points using the AAM fitting process. In addition, the performance of AAM is affected by face movement, face area illumination, and background texture. Thus, we roughly define five wrinkle areas based on eyes and facial box position, as shown in Figure 10, rather than nine areas, as in [7]. Compared to the nine areas in [7], the four regions (those between the eye corner and left (or right) face boundary and those between the lip corner and left (or right) face boundary) are not used in our system because accurate face boundary detection is not guaranteed without an AAM.

**Figure 11:**
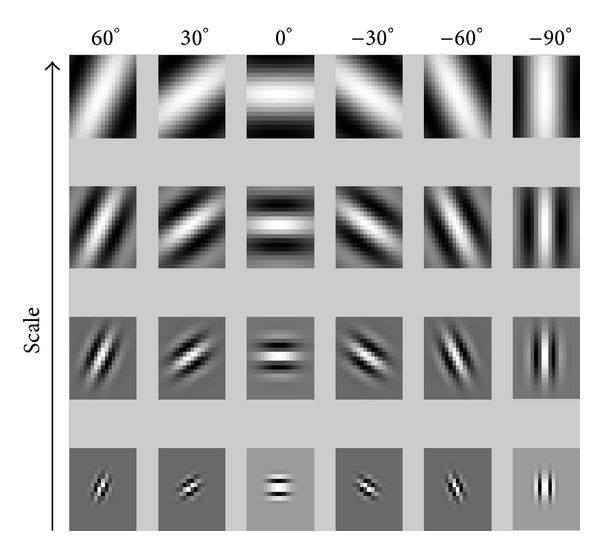
Example of Gabor filters with four scales and six directions.

For each local region, the features are extracted using Gabor filtering. The two-dimensional (2D) Gabor filter in the spatial domain is defined as follows [7, 20]:

(3) $$\begin{array}{c} g\left(x,y\right)=\left(\frac{1}{2\pi \sigma_{x}\sigma_{y}}\right)\exp\left[-\frac{1}{2}\left(\frac{x^{2}}{{\sigma_{x}}^{2}}+\frac{y^{2}}{{\sigma_{y}}^{2}}\right)+2\pi jWx\right], \end{array}$$

where *σ*_*x*_ and *σ*_*y*_ are the standard deviations of *g*(*x*, *y*) on the *x*- and *y*-axes, respectively, and *W*  is the radial frequency of a sinusoid. In our method, only the real part of Gabor filter is used for fast processing. We obtained the filter coefficients experimentally considering the previous research [7]. According to the human age, the appearance (direction and thickness) of wrinkle feature in five selected local areas is different. With a young person, the thickness of wrinkle is narrow whereas it is wider when the person becomes older. Therefore, in order to extract the wrinkle feature of various thickness and directions, we used the Gabor filter with four scales and six directions, as shown in Figure 11. The mean and standard deviation of the Gabor filtering response are used as the wrinkle features. Consequently, the local feature using Gabor filtering is a vector in 240-dimensional space (5 regions × 4 scales × 6 directions × 2 features).

#### 2.3.4. Feature Fusion and Age Estimation Using SVR

With the global features obtained using MLBP and the local features obtained using Gabor filtering, a final feature vector is constructed by concatenating the two normalized features, using z-score normalization [7] as shown in

(4) $$\begin{array}{c} f_{i}^{\mathrm{norm}}=\frac{f_{i}-\mu_{i}}{\sigma_{i}}, \end{array}$$

where *f*_*i*_^norm⁡^ stands for the *i*th normalized feature vector of the original feature*f*_*i*_. *μ*_*i*_ and *σ*_*i*_ are the mean and standard deviation of the distribution of *f*_*i*_, respectively. In our research, the values of *i* are 1 and 2 in case of MLBP and Gabor filtering features, respectively. After normalization, the combined feature vector *f* is easily obtained by concatenating these two normalized vectors together:

(5) $$\begin{array}{c} f=\left[f_{1}^{\mathrm{norm}},f_{2}^{\mathrm{norm}}\right]. \end{array}$$

This feature vector is inputted to the SVR machine implemented using LibSVM software [21]. The optimal SVR kernel with its parameters is determined experimentally using training data, from which we can obtain the best relation between the extracted feature vector *f* and the ground-truth age of human in input image.

## 3. Experimental Result

### 3.1. Database

**Figure 12:**
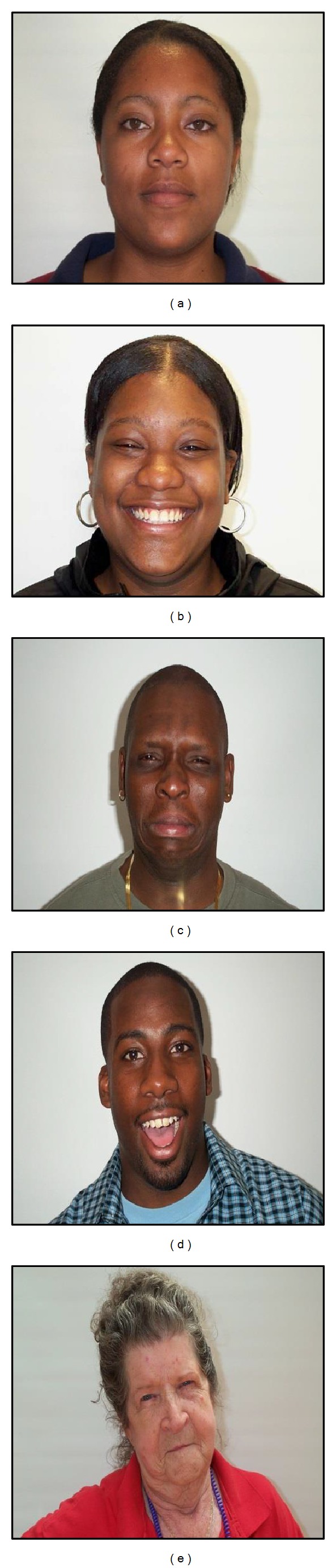
Example images in PAL database with varied genders and facial expressions: (a) female-neutral expression, (b) female-happy expression, (c) male-sad expression, (d) male-surprised expression, and (e) female-annoyed expression.

For experiments, a lifespan aging database (PAL database) that contains both gender and facial expression was used [22, 23]. The PAL database was obtained from 576 people of 18 to 93 years old, including Caucasians, African-Americans, and others. We used 1,045 of these images, excluding those for which face detection failed. These images include 429 males and 616 females, distributed into eight general facial expressions such as angry, annoyed, disgusted, grumpy, happy, neutral, sad, and surprised.  Figure 12 shows some example PAL database images with varied genders and facial expressions.

### 3.2. Experimental Result

#### 3.2.1. Performance Evaluation of Our Age Estimation System

To measure the accuracy of our age estimation system, we chose the mean absolute error (MAE), widely used in previous research and shown in [7, 9, 13]

(6) $$\begin{array}{c} \text{MAE}=\frac{1}{N}\overset{N}{\underset{k=1}{\sum }}\left|a_{k}^{\prime }-a_{k}\right|, \end{array}$$

where *N* is the total number of images in the testing data set, *a*_*k*_ is the ground-truth age of the *k*th image, and *a*_*k*_′ is its estimated one. It can be inferred from (6) that the smaller MAE value indicates better age estimation performance.

To measure the performance of our age estimation system, we performed 2-fold cross-validation. In each experiment we randomly divide the entire database into two parts: learning and testing databases. The PAL aging database has been widely used for age estimation in previous researches. However, this database does not provide the identity information. Instead, only the information of race, gender, age, and facial expression is associated with the name of image. Without the identity information, it is very difficult to separate the dataset into two parts containing different individuals. Therefore, we randomly divided the database into learning and testing twice in order to perform the experiments based on 2-fold cross-validation. All the parameters with kernels are trained with the learning database, and the MAE is measured with the testing database. In the second trial, the learning and testing databases are again determined randomly, and the procedure is repeated. From this procedure, two MAEs are obtained, with the average value of the two MAEs calculated as the final MAE.

For the first experiment, we compared the accuracies of our age estimation system based on single-level LBP and MLBP. For the experiments, the LBP accuracies with various *R* values (in the range of 1 to 5) and *P* values (8, 12, and 16) for (2) were compared. As shown in Figure 5, the extracted texture features by LBP operator are different according to various values of *R* and *P*. In order to obtain the optimal values of *R* and *P* (with which, the MAE of age estimation is minimized), we performed the experiments with the various values of *R* and *P*. In addition, we compared the accuracies of using a rectangular or square shape for each subblock of Figures 8 and 9, according to the various numbers of subblocks. As shown in Table 1, the MLBP-based method outperforms the LBP-based method, and we used the MLBP-based method for our age estimation system.

In the next experiment, we compared the MAEs using only MLBP, only Gabor filtering, and the proposed method combining the two. In addition, we compared the MAE using the proposed method to that obtained in previous researches [9, 13]. In Table 2, the method using only MLBP with square block division is from [13]. The extracted LBP feature vectors can be different according to the shapes of each subblock, which can affect the accuracy of age estimation. So, we measured the MAEs according to the shapes of each subblock. The rectangular block means that the height and width of the subblock are different whereas the squared one indicates that the height and width of the subblock are the same. As shown in Table 2, the accuracy using the proposed method is higher than that in the other cases and in previous research.

In our method, Gabor filtering is used to extract the local wrinkle feature. As the human becomes older, the wrinkle feature appears, and the strength (depth) of wrinkle feature can be used for the discrimination of different ages [7]. Although the accuracy of age estimation by the Gabor filtering is worse than that by MLBP method, the MAE by combining these two methods is lower than that by using only MLBP method or Gabor filtering as shown in Table 2.

We measured the difference between the MAEs by our method and that by previous one [13] of Table 2 based on effect size in descriptive statistics. In statistics, the power of a measured phenomenon can be evaluated based on effect size, and as a descriptive statistic, the effect size has been widely used [24, 25]. Based on the previous research [26], the values of 0.2, 0.5, and 0.8 can be defined as small, medium, and large for Cohen's *d* value, respectively.

In Table 3, Cohen's *d* show the difference between two means divided by a standard deviation of the data. The effect size of 0.2 to 0.3, around 0.5, and 0.8 to infinity (based on Cohen's *d* value) can be “small,” “medium,” and “large” effect, respectively [24, 25]. Since all the Cohen's *d* values of Table 3 are less than 0.2, we can find that there exists a difference between the MAEs by our method and previous one [13] as a small effect size.

**Figure 13:**
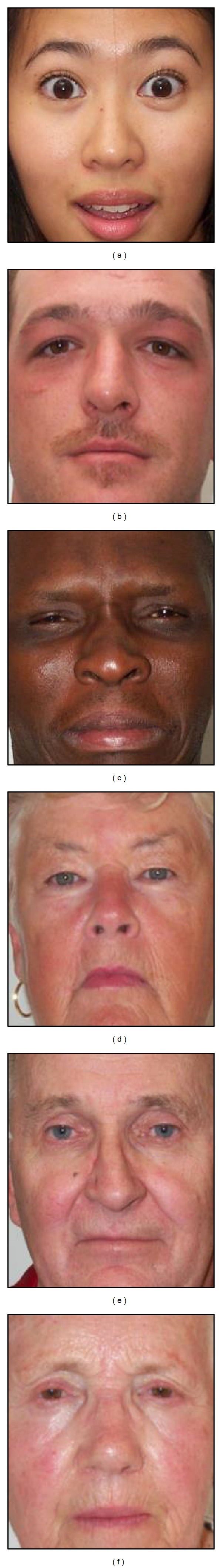
Examples of estimated and ground-truth ages: (a) ground-truth age of 19, estimated age of 19, (b) ground-truth age of 32, estimated age of 31, (c) ground-truth age of 41, estimated age of 40, (d) ground-truth age of 65, estimated age of 65, (e) ground-truth age of 72, estimated age of 72, and (f) ground-truth age of 81, estimated age of 80.

In Figure 13, we show some examples of estimated ages using the proposed method and ground-truth.

**Figure 14:**
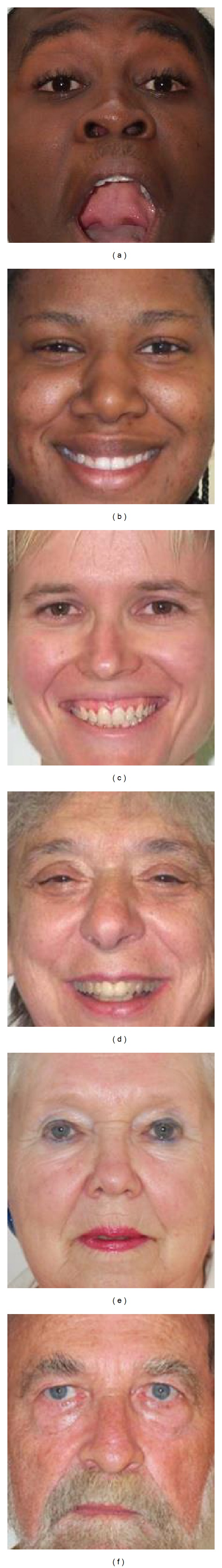
Examples of estimated and ground-truth ages with a large error: (a) ground-truth age of 19, estimated age of 30, (b) ground-truth age of 22, estimated age of 32, (c) ground-truth age of 29, estimated age of 37, (d) ground-truth age of 66, estimated age of 72, (e) ground-truth age of 75, estimated age of 68, and (f) ground-truth age of 64, estimated age of 74.

In addition, we include the examples (where the errors of age estimation are large) as shown in Figure 14. The errors of Figures 14(a)–14(d) are caused by the facial expression. Those of Figures 14(e) and 14(f) are due to the facial makeup and mustache, respectively.

#### 3.2.2. Study of Effects of Facial Expression and Gender on Age Estimation

In the next experiments, we study the effects of facial expression and gender on the estimation system using our proposed age estimation method of Section 2.3 and Table 2. Although the number of images used in the above experiments is 1,045, as explained in Section 3.1, the images of angry, grumpy, and disgusted are not included for the next experiment because these images are too few. Consequently, the images of five expressions are used for the experiments, as shown in Table 4. As explained in Section 2.1, the facial expression databases are separated manually from the whole PAL database based on facial expression markers attached to an image's name.

As shown in Table 5, the average MAE (7.226) of age estimation with facial expression preclassification is greater than that (6.528) without preclassification. Only the neutral facial expression database produces a better estimation result compared to that of the whole PAL database. There are several reasons why the MAEs with facial expression preclassification are greater than those without preclassification, and these are explained in Section 4.

Next, we study the effects of gender (male and female) on the estimation system. All 1,045 images are used for the experiments, as shown in Table 6. As explained in Section 2.1, the gender databases are separated manually from the whole PAL database based on gender markers attached to an image's name. In Tables 5 and 6, the average MAEs were calculated by weighting each MAE with number of samples of each class.

As shown in Table 7, the average MAE (6.323) of age estimation with gender preclassification is less than that (6.528) without preclassification. In addition, the male database MAEs are lower than those of the female. These results are explained in Section 4.

As the next experiment, we compared the accuracy of our method to that in [27] which used octet-based MLBP method. With PAL database, we show the mean absolute errors (MAEs) of age estimation by the previous octet-based MLBP method [27] as shown in Table 8. In order to obtain the optimal sizes of image and LBP operator, we performed the experiments according to various sizes of facial image and LBP operator. As shown in Table 8, the lowest MAE is 12.693 years old which is much larger than that (6.581) by our method as shown in Table 1. Therefore, we find that our method outperforms the previous octet-based MLBP method.

## 4. Discussion

In Table 5, we showed the comparative estimation results of facial expression databases and the whole PAL database. The best performance was obtained for the neutral expression database with an MAE of 6.360 years old. The estimation results of other facial expression databases are unsatisfactory compared to those of the whole PAL database. Intuitively, we would expect that the MAEs of the whole PAL database would be greater than those of the facial expression database because the variation factors by facial expression in the whole PAL database also affect age estimation performance. However, by dividing the whole PAL database into the five facial expression databases, the number of images for learning in each expression database becomes so small that the learning database cannot reflect the characteristics of the testing database. However, the number of the neutral database is greater than that of the others, and the consequent learning of the neutral database can reflect testing database characteristics sufficiently. Consequently, the facial expression database MAEs (except for the neutral database) are greater than those of the whole PAL database.

The comparative estimation results of gender databases and the whole PAL database are presented in Table 7. Compared to those of the whole PAL database, the average gender database MAE is less than that of the whole PAL database, and we can confirm that gender preclassification can affect age estimation accuracy. However, the MAEs of the female database are greater than that of the whole PAL database, whereas that of the male database is much less than those of the other cases. The reason this occurs is as follows.

**Figure 15:**
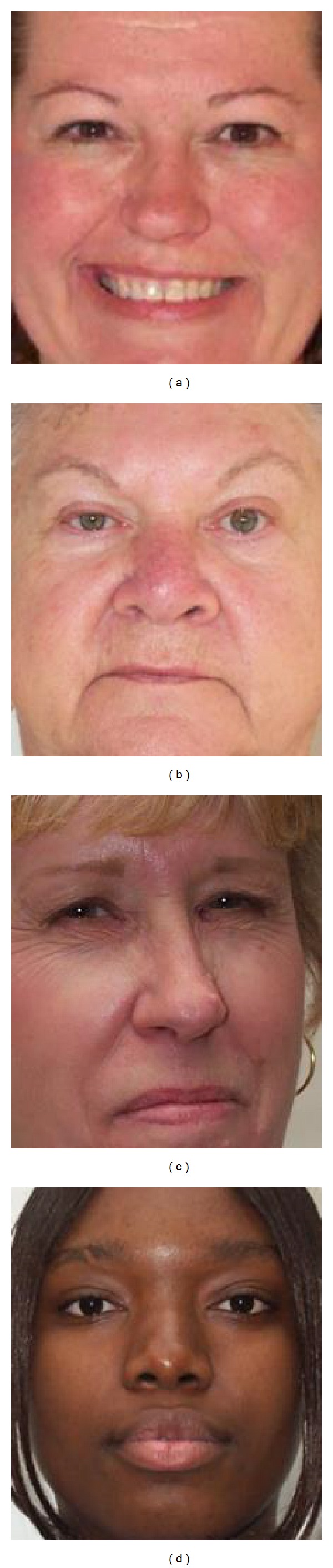
Example of estimation results of the female database: (a) ground-truth age of 42, estimated age of 43, (b) ground-truth age of 70, estimated age of 70, (c) ground-truth of 53, estimated age of 72, and (d) ground-truth of 20, estimated age of 36.

The female database includes wider hairstyle variation than the male, as shown in Figures 15(c) and 15(d). In addition, variations caused by cosmetics are wider in the female database. Thus, these variations of learning data in the female database cannot be trained adequately using only a female database with a small number of images; hence, the MAE of the whole PAL database, which was trained using more learning samples, can be smaller. Although the number of images in the male database is less than that in the female database, the variations in the male database are narrower; hence, the consequent learning images of the male database can reflect testing database characteristics adequately. Thus, the MAEs of the male database are less than those of the female and whole PAL databases.

There have been a lot of previous methods for automatic recognition of gender and facial expression. Therefore, the errors of preclassification are different according to the specific recognition method and the consequent effect of gender and facial expression on age estimation changes. Since we try to analyze only the effect of gender and facial expression on age estimation system without other factors, we used the method of manual preclassification of gender and facial expression in our experiment.

In our experiment, among a total of 1,046 facial images in PAL aging database, there was only 1 image where face detection failed using AdaBoost method. This error image was not used for age estimation because the main point of our research is not face detection but age estimation. In addition, in real applications, if the system fails in detecting the human's face from input image, the further step of age estimation is not performed, but the step of recapturing is repeated until the face detection is successful. Therefore, the 1 error image among 1,046 ones can be neglected in the real application of age estimation.

## 5. Conclusion

In this paper, we proposed a new age estimation method based on a combination of MLBP, Gabor filtering, and SVR. The experimental results showed that the proposed age estimation method outperforms previous methods by producing a better estimation result. Using the proposed age estimation method, we investigated the effects of gender and facial expression on estimation performance. We confirmed that gender and facial expression affect age estimation only if the system can be trained adequately with a large number of images. In future work, we plan to enhance the age estimation performance of our method using the scheme of assigning adaptive weights to MLBP features of each subblock based on neural networks or fuzzy systems. In addition, other effects of race, image resolution, and focusing condition on the performance of age estimation will be studied.

## Figures and Tables

**Table 1:** Comparisons of MAEs of MLBP method to those of single-level LBP method (unit: years old).

Database and shape of subblock	Single-level LBP	MLBP
Testing database 1		
Rectangular block division	6.981	6.351
Squared block division	7.125	6.536
Testing database 2		
Rectangular block division	7.531	7.322
Squared block division	7.345	6.625
Average MAE		
Rectangular block division	7.256	6.837
Squared block division [13]	7.235	**6.581**

**Table 2:** The comparisons of MAEs using only MLBP, only Gabor filtering, and the proposed method combining the two methods (MLBP and Gabor filtering) (unit: years old).

Whole PAL database	Using only MLBP	Using only Gabor filtering	Proposed method
Testing database 1			
Rectangular block division	6.351	11.774	6.247
Squared block division	6.536		6.513
Testing database 2			
Rectangular block division	7.322	12.042	7.176
Squared block division	6.625		6.542
Average MAE			
Rectangular block division	6.837	11.908	6.712
Squared block division	6.581		**6.528**
Previous research [9]		8.44	
Previous research [13]		6.581	

**Table 3:** The measured Cohen's *d* between the MAEs by our method and previous one [13].

	Testing database 1	Testing database 2	Average
Cohen's *d*	0.004183	0.014779	0.009481
Effect size	Small	Small	Small

**Table 4:** The number of images used for the comparative experiments with/without the preclassification of facial expression.

Database	Number of learning images	Number of testing images
Facial expression database		
Annoyed	21	19
Happy	130	128
Neutral	291	289
Sad	32	32
Surprised	39	38
Total	**513**	**506**
Whole PAL database	523	522

**Table 5:** Comparison of MAEs with and without the preclassification of facial expression (unit: years old).

Database	Using only MLBP	Using only Gabor filtering	Proposed method
Facial expression database			
Annoyed	6.00	10.526	6.605
Happy	8.121	12.168	8.102
Neutral	6.396	11.644	6.360
Sad	10.063	12.484	10.641
Surprised	8.645	10.671	8.290
Average MAE	7.218	11.715	**7.226**
Whole PAL database	6.581	11.908	6.528

**Table 6:** The number of images used for the comparative experiments with/without the preclassification of gender.

Database	Number of learning images	Number of testing images
Gender database		
Male	215	214
Female	309	307
Total	**524**	**521**
Whole PAL database	523	522

**Table 7:** Comparison of MAEs with and without the preclassification of gender (unit: years old).

Database	Using only MLBP	Using only Gabor filtering	Proposed method
Gender database			
Male	5.796	11.086	5.783
Female	6.816	12.350	6.699
Average MAE	6.397	11.831	**6.323**
Whole PAL database	6.581	11.908	6.528

**Table 8:** MAEs measured by the previous MLBP method (unit: years old).

Size of facial image	Size of LBP operator		
	3 × 3 operator	5 × 5 operator	7 × 7 operator
64 × 64	16.617	14.656	13.966
96 × 96	15.584	13.503	13.612
128 × 128	16.784	12.878	**12.693**
160 × 160	16.803	13.376	12.730
192 × 192	17.711	13.809	13.119
220 × 250	18.365	13.460	12.813
